# Lung-protective ventilation worsens ventilator-induced diaphragm atrophy and weakness

**DOI:** 10.1186/s12931-020-1276-7

**Published:** 2020-01-10

**Authors:** Xian-Long Zhou, Xiao-Jun Wei, Shao-Ping Li, Hao-Li Ma, Yan Zhao

**Affiliations:** 1 Emergency Center, Zhongnan Hospital of Wuhan University, 169 Donghu Road, Wuhan, 430071 Hubei China; 2 Department of Biological Repositories, Zhongnan Hospital of Wuhan University, 169 Donghu Road, Wuhan, 430071 Hubei China

**Keywords:** Lung–protective ventilation, Mechanical ventilation, Diaphragm dysfunction, PGC–1α, Oxidative stress

## Abstract

**Background:**

Lung–protective ventilation (LPV) has been found to minimize the risk of ventilator–induced lung injury (VILI). However, whether LPV is able to diminish ventilator–induced diaphragm dysfunction (VIDD) remains unknown. This study was designed to test the hypothesis that LPV protects the diaphragm against VIDD.

**Methods:**

Adult male Wistar rats received either conventional mechanical (tidal volume [V_T_]: 10 ml/kg, positive end–expiratory pressure [PEEP]: 2 cm H_2_O; CV group) or lung-protective (V_T_: 5 ml/kg, PEEP: 10 cm H_2_O; LPV group) ventilation for 12 h. Then, diaphragms and lungs were collected for biochemical and histological analyses. Transcriptome sequencing (RNA–seq) was performed to determine the differentially expressed genes in the diaphragms between groups.

**Results:**

Our results suggested that LPV was associated with diminished pulmonary injuries and reduced oxidative stress compared with the effects of the CV strategy in rats. However, animals that received LPV showed increased protein degradation, decreased cross–sectional areas (CSAs) of myofibers, and reduced forces of the diaphragm compared with the same parameters in animals receiving CV (*p* < 0.05). In addition, the LPV group showed a higher level of oxidative stress in the diaphragm than the CV group (*p* < 0.05). Moreover, RNA–seq and western blots revealed that the peroxisome proliferator–activated receptor γ coactivator–1alpha (PGC–1α), a powerful reactive oxygen species (ROS) inhibitor, was significantly downregulated in the LPV group compared with its expression in the CV group (*p* < 0.05).

**Conclusions:**

Compared with the CV strategy, the LPV strategy did not protect the diaphragm against VIDD in rats. In contrast, the LPV strategy worsened VIDD by inducing oxidative stress together with the downregulation of PGC–1α in the diaphragm. However, further studies are required to determine the roles of PGC–1α in ventilator-induced diaphragmatic oxidative stress.

## Introduction

Increasing evidence has demonstrated that prolonged mechanical ventilation (MV) is a dangerous intrusion that has the potential to harm the lungs, a condition known as ventilator–induced lung injury (VILI) [1]. So–called ‘lung–protective’ ventilator settings aimed at the prevention of VILI have been introduced, and the concept of lung–protective ventilation (LPV), defined as ventilation with lower tidal volume (V_T_)(≤ 5 ml/kg) and higher positive end–expiratory pressure (PEEP) (≥ 10 cm H_2_O), has been well established among patients with normal lungs [2] and those with acute lung injury (ALI) [3]. Currently, the LPV strategy has been routinely used for mechanically ventilated patients because the use of LPV was associated with improved clinical outcomes in terms of improved respiratory mechanics, reduced pulmonary complications, and decreased mortality [4–6]. However, it has been demonstrated that LPV (≥ 6 h) also induces rapid diaphragm disuse atrophy and weakness, which has been referred to as ventilator–induced diaphragm dysfunction (VIDD) [7]. In addition, increasing evidence has indicated that VIDD is a major complication of MV and that its development is tightly associated with poor clinical outcomes in critically ill patients due to weaning failure and increased time of MV [7–10]. Although the molecular mechanism of VIDD has not yet been completely understood, various manifestations of VIDD have been linked to excessive reactive oxygen species (ROS) production and oxidative stress [11]. In addition, the development of VIDD is largely prevented when mitochondria–targeted or non–selective antioxidants have been administered to the animals at the onset of MV [12, 13]. Some evidence suggests that peroxisome proliferator–activated receptor γ coactivator–1alpha (PGC–1α), a key regulator of ROS production [14], can protect skeletal muscle against atrophy [15] and that the downregulation of PGC–1α contributes to the production of ROS and oxidative stress during skeletal disuse atrophy [16]. However, to the best of our knowledge, the roles of PGC–1α in the pathogenesis of VIDD have not yet been reported.

Recently, the concept of a diaphragm–protective ventilation strategy has arisen from the notion that diaphragm weakness results from either unloading or excessive loading. Based on an understanding of various mechanisms of diaphragm myotrauma during ventilation, it has been demonstrated that VIDD can be prevented by adaptive or partial-support ventilation [17–19]. Currently, some believe that lung protection is the primary priority because of strong evidence indicating its benefits for patient outcomes, which means a diaphragm–protective ventilation strategy should be, first, lung protective. It is commonly accepted that LPV is able to reduce pulmonary injuries compared with the injuries associated with a conventional mechanical ventilation (CV) strategy with high V_T_ and low PEEP [20]. Animal experiments and clinical trials have demonstrated that the LPV strategy protects the lungs against ventilator–induced lung injury through the downregulation of oxidative stress [21, 22]. However, a very recent study found that ventilation with PEEP results in longitudinal atrophy of diaphragm fibers [23], which suggested that high PEEP possibly acts as a risk factor for the structural damage of diaphragms and the development of weakness. Moreover, it has been reported that low V_T_ (6 ml/kg) ventilation improved diaphragm muscle force generation without histological changes compared with the effects of high V_T_ (10 ml/kg) ventilation after 6 h of ventilation [24]. Therefore, whether LPV is more beneficial than CV for the protection of the diaphragm against VIDD remains uncertain. In the present study, we aimed to test the hypothesis that the LPV (low V_T_/high PEEP), but not the CV (high V_T_/low PEEP), strategy protects the diaphragm against VIDD and to investigate the underlying mechanism in a rat model of MV.

## Methods and materials

### Animals

Fifteen male Wistar rats, weighing 400 to 450 g, were purchased from the Charles River Laboratories (Beijing, China). Animals were kept under controlled conditions under a 12:12 light–dark cycle. Water and food were provided ad libitum. Animal experiments were performed in accordance with the Guide for the Care and Use of Laboratory Animals.

### Grouping

Animals were randomly assigned into 3 groups: (1) a control group (CON, *n* = 5): animals underwent surgical exposure of the trachea but not tracheotomy. Then, animals breathed spontaneously for 12 h; (2) a conventional mechanical ventilation group (CV, *n* = 5): animals received MV for 12 h with a V_T_ of 10 ml/kg and a PEEP of 2 cm H_2_O; and (3) a lung–protective ventilation group (LPV, *n* = 5): animals received MV for 12 h with a V_T_ of 5 ml/kg and a PEEP of 10 cm H_2_O. At the end of the experiment, animals were sacrificed by blood depletion under anesthesia for sample collections.

### Mechanical ventilation

The animal MV model was established as previously described [25]. Briefly, animals were tracheostomized and connected to a small animal ventilator (VentElite, Harvard Apparatus; Cambridge, MA, USA) after anesthesia with sodium pentobarbital (40 mg•kg^− 1^ body weight, i.p.). Breathing air was humidified and enriched with oxygen. To maintain arterial PaO_2_ at 80 to 100 mmHg and PaCO_2_ at 35 to 45 mmHg, the respiration rate and oxygen supply were adjusted according to the results of blood gas analysis. The mean arterial pressure (MAP) was monitored at the tail using tail cuff plethysmography (BP–2010, Softron, Japan). The right jugular vein was infused with normal saline (1 ml•kg^− 1^•h^− 1^) and pentobarbital sodium at a rate of 10 mg•kg^− 1^•h^− 1^. Body temperature was maintained at 37 °C by a homoeothermic blanket.

### Arterial blood gases and lactate analysis

Arterial pH, PaO_2_, PaCO_2_, HCO_3_^−^, base excess and serum lactate levels were determined using an i–STAT1Analyzer (Abbott, Kyoto, Japan).

### Histological analysis

Paraffin–embedded pulmonary tissues were stained with hematoxylin and eosin (H&E). The degrees of lung damage were determined by a modified VILI score, as previously reported [26]. Tissue sections were evaluated by two independent observers in a blinded manner based on 3 following aspects: (1) alveolar wall thickness; (2) infiltration or aggregation of inflammatory cells; and (3) hemorrhage. Each item was graded according to a five–point scale: 0 = no injury, 1 = mild injury, 2 = moderate injury, 3 = severe injury and 4 = maximal damage.

### Immunofluorescence staining

Immunofluorescence staining was performed on frozen tissues to evaluate the CSA of muscle fibers. NOQ7.5.4D antibody and MY–32 antibody (Abcam) were used for the identification of the slow and fast myosin heavy chains, respectively. Anti–laminin (Abcam) was used to outline the myofibers.

### Muscle force measurements

Muscle contractile properties were measured as previously described [27]. Each muscle strip was rapidly mounted in a tissue chamber containing Krebs–Henseleit solution (27 °C, pH 7.4), which was bubbled with a gas mixture of 95% O_2_–5% CO_2_. Muscle extremities were held in spring clips and attached to an electromagnetic force transducer for the measurements.

### Measurements of inflammatory cytokines

Inflammatory cytokines, including tumor necrosis factor (TNF)–α, interleukin (IL)–6 and IL–1β, were detected using commercial enzyme–linked immunosorbent assay kits (HS Quantikine; R&D Systems, Minneapolis, MN, USA) according to the manufacturer’s instructions.

### Assessment of pulmonary and diaphragmatic oxidative stress

The lipid peroxidation end–product 4–hydroxynonenal (4–HNE) is generated in tissues during oxidative stress. In the present study, the expression of 4–HNE was measured using western blot assays as a marker of pulmonary and diaphragmatic oxidative stress.

### RNA–seq

Total RNA was extracted using the mirVana miRNA Isolation Kit (Ambion) following the manufacturer’s protocol. RNA integrity was evaluated using the Agilent 2100 Bioanalyzer (Agilent Technologies, USA), and the samples with an RNA Integrity Number (RIN) ≥ 7 were subjected to the subsequent analysis. The libraries were constructed using TruSeq Stranded Total RNA with Ribo–Zero Gold (Illumina) according to the manufacturer’s instructions. Then, these libraries were sequenced on the Illumina sequencing platform (HiSeqTM 2500), and 150 bp/125 bp paired–end reads were generated.

Raw reads generated during high-throughput sequencing were fastq format sequences. Trimmomatic software [28] was first used to obtain high-quality clean reads by removing adapter sequences and filtering out low-quality bases and low-quality reads. Then, hisat2 [29] software was used to align clean reads to the reference rat genome, and Stringtie software [30] was used to assemble the reads into transcripts. After aligning the sequencing reads of each sample with the sequence of mRNA transcript sequences by bowtie2 and using eXpress to perform gene quantitative analysis, the FPKM value and count value (the number of reads for each gene in each sample) were obtained. The estimateSizeFactors function of the DESeq [31] R package was used to normalize the counts, and the nbinomTest function was used to calculate *p* values and fold change values for the difference comparison. Moreover, a heatmap was built by using the pheatmap *R* package.

### Western–blot assay

Equal amounts of proteins were resolved by SDS–PAGE, and the proteins were transferred to Hybond ECL membranes (Amersham, Buckinghamshire, UK). The membranes were incubated with primary antibodies, including LC3B–II/I, Atrogin–1, MuRF–1, PGC–1α, and 4–HNE (Abcam, USA), at 4 °C overnight. After washing with TBST, the membranes were probed with secondary antibodies and visualized using an enhanced chemiluminescence system (Kodak, Rochester, NY, USA). GAPDH was used as a loading control.

### Statistical analysis

Data are expressed as numbers, percentages, medians [25th and 75th percentile] or the means ± SDs. The comparison of means was performed using one–way analysis of variance. Comparisons between two groups were performed by unpaired Student’s *t*–test. VILI scores were compared using the Steel–Dwass test followed by the Kruskal–Wallis test. Percentages were compared using Fisher’s exact test. All statistical analyses were performed using *R* packages. All *p* values were two-tailed, and a *p* value less than 0.05 was considered significant.

## Results

### Systemic responses to CV and LPV

The differences in pH values and serum lactate levels were insignificant between the LPV and CV groups. The PaCO_2_ levels in the CV group were nonsignificantly lower than those in the LPV group. In addition, no significant differences were observed in the MAPs between the LPV and CV groups. Importantly, no significant differences were observed in blood gases, lactate levels or MAPs between the CON and the mechanically ventilated groups (LPV and CV) (*p* > 0.05, respectively). The data are summarized in Table 1.

**Table 1:** Summary of ventilator settings, blood gas analysis and vital signs

Group	CON (*n* = 5)	LPV (*n* = 5)	CV (*n* = 5)
Ventilator settings			
Tidal volume (ml/kg)	N/A	5	10
PEEP (cm H_2_O)	N/A	10	2
Arterial blood gas analysis			
Arterial pH	7.37 ± 0.06	7.42 ± 0.04	7.47 ± 0.03
PaCO_2_ (mmHg)	32 ± 3	39 ± 12	27 ± 6
PaO_2_ (mmHg)	95 ± 8	98 ± 5	113 ± 20
HCO_3_^−^ (mmol/L)	26.9 ± 4.3	24.7 ± 6.3	19.5 ± 4.2
Lactate (mmol/L)	1.13 ± 0.49	1.70 ± 1.40	1.73 ± 0.32
Vital signs			
MAP (mmHg)	90 ± 15	84 ± 17	86 ± 11
Heart rate (beats/min)	392 ± 28	394 ± 23	397 ± 34
Rectal temperature (°C)	37.0 ± 0.1	37.1 ± 0.1	37.1 ± 0.2

Data are expressed as number or mean ± SD; *N/A* not applicable, *PEEP* positive end–expiratory pressure, *MAP* mean arterial pressure

### Pulmonary injuries in healthy rats after CV and LPV

**Fig. 1:**
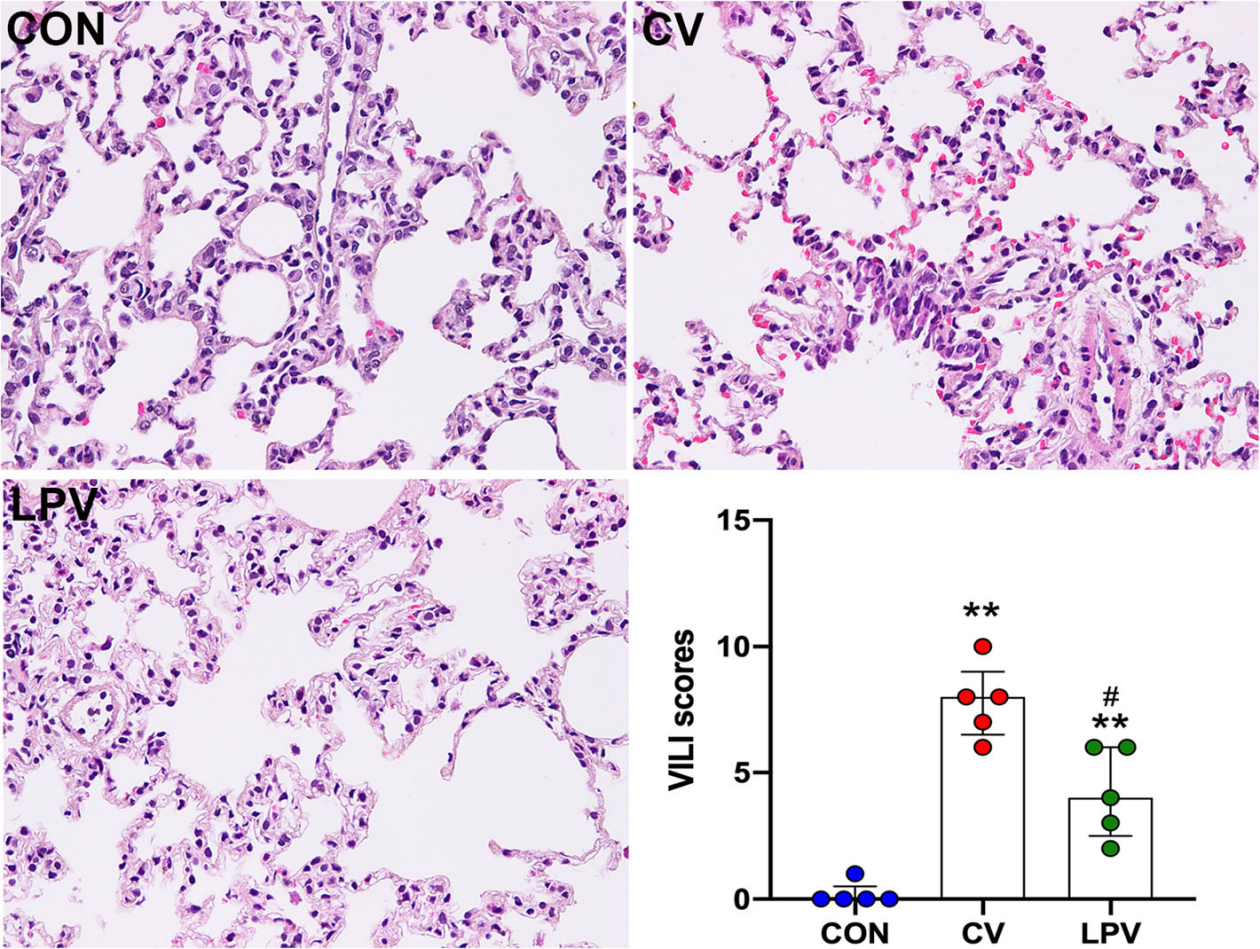
LPV protected lungs against VILI. H&E staining (400×) showed that either CV or LPV induced pulmonary injuries in rats, with significantly higher VILI scores than that in the CON group. In addition, pulmonary injuries in the CV group were greater than that in the LPV group as reflected by higher VILI scores; **p < 0.01 vs. CON group; ^#^*p* < 0.05 vs. CV group

Histological analysis was performed under a light microscope after H&E staining (Fig. 1). The median VILI scores in the CV and LPV groups were significantly higher than those in the CON group (8 [7, 9], 4 [3, 6] vs. 0 [0, 1]; *p* < 0.01, respectively). In addition, VILI scores in the LPV group were significantly lower than those in the CV group (4 [3, 6] vs. 8 [7, 9], *p* = 0.0238). The damage severity distribution of each item in all groups is summarized in Additional file 1: Table S1. The percentage of severe/maximal damage in alveolar wall thickness, inflammatory cell infiltration and hemorrhage in the CV group were significantly higher than those in the LPV group (*p* < 0.05, respectively). These results suggested that both LPV and CV strategies induced pulmonary injury, but the LPV strategy reduced morphological damage.

### Diaphragm atrophy and weakness after CV and LPV

**Fig. 2:**
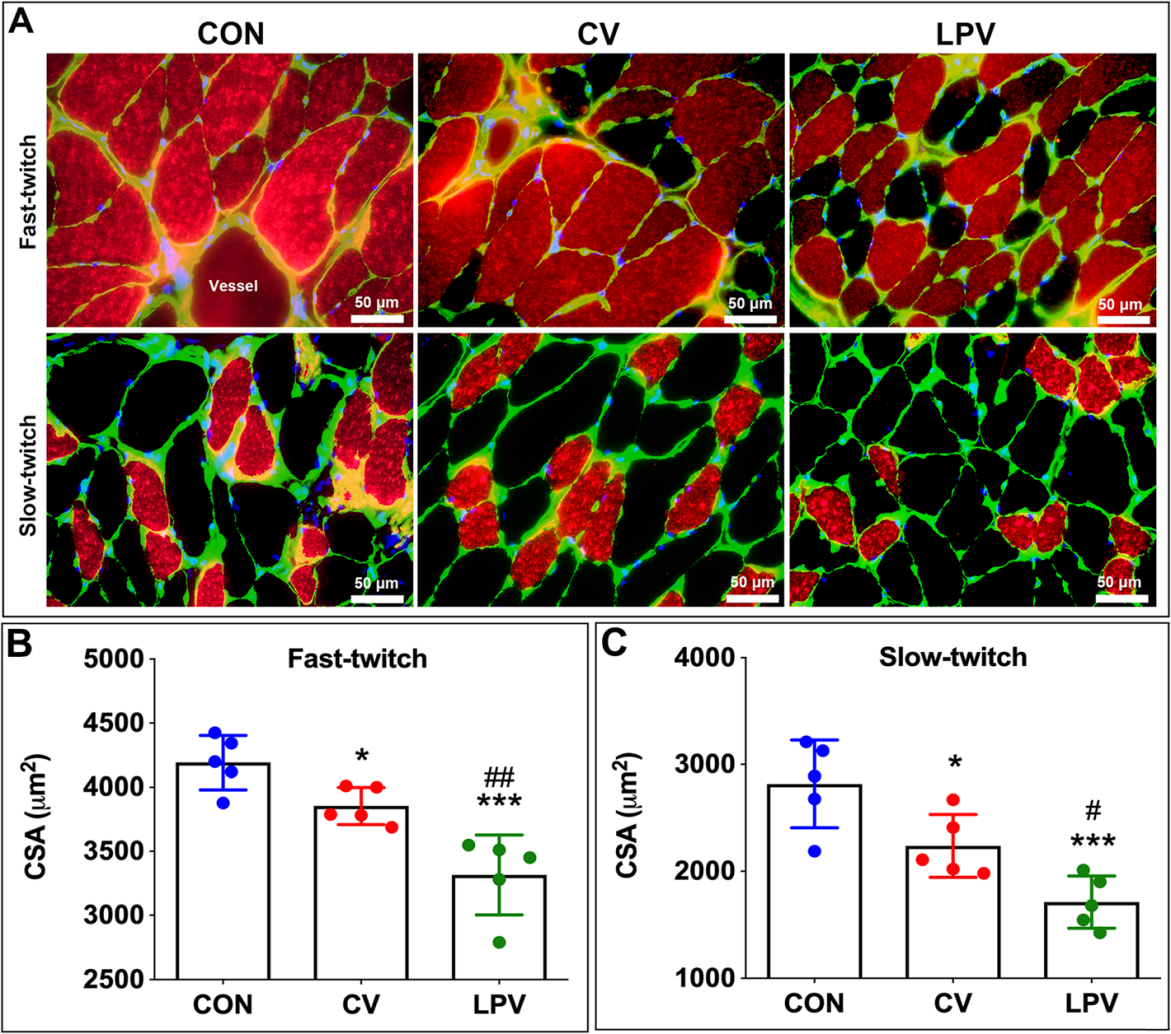
LPV reduced myofiber CSA in the diaphragm. (**a**), immunofluorescence staining for slow–twitch and fast–twitch fibers; The cross–sectional areas (CSA) of both slow–twitch (**b**) and fast–twitch (**c**) myofibers in the LPV and the CV groups were significantly decreased as compared with the CON group; In addition, CSA of diaphragm myofibers in the LPV group were significantly lower than that in the CV group. *p < 0.05, ***p < 0.001 vs. CON group; ^#^*p* < 0.05, ^##^*p* < 0.01 vs. CV group

**Fig. 3:**
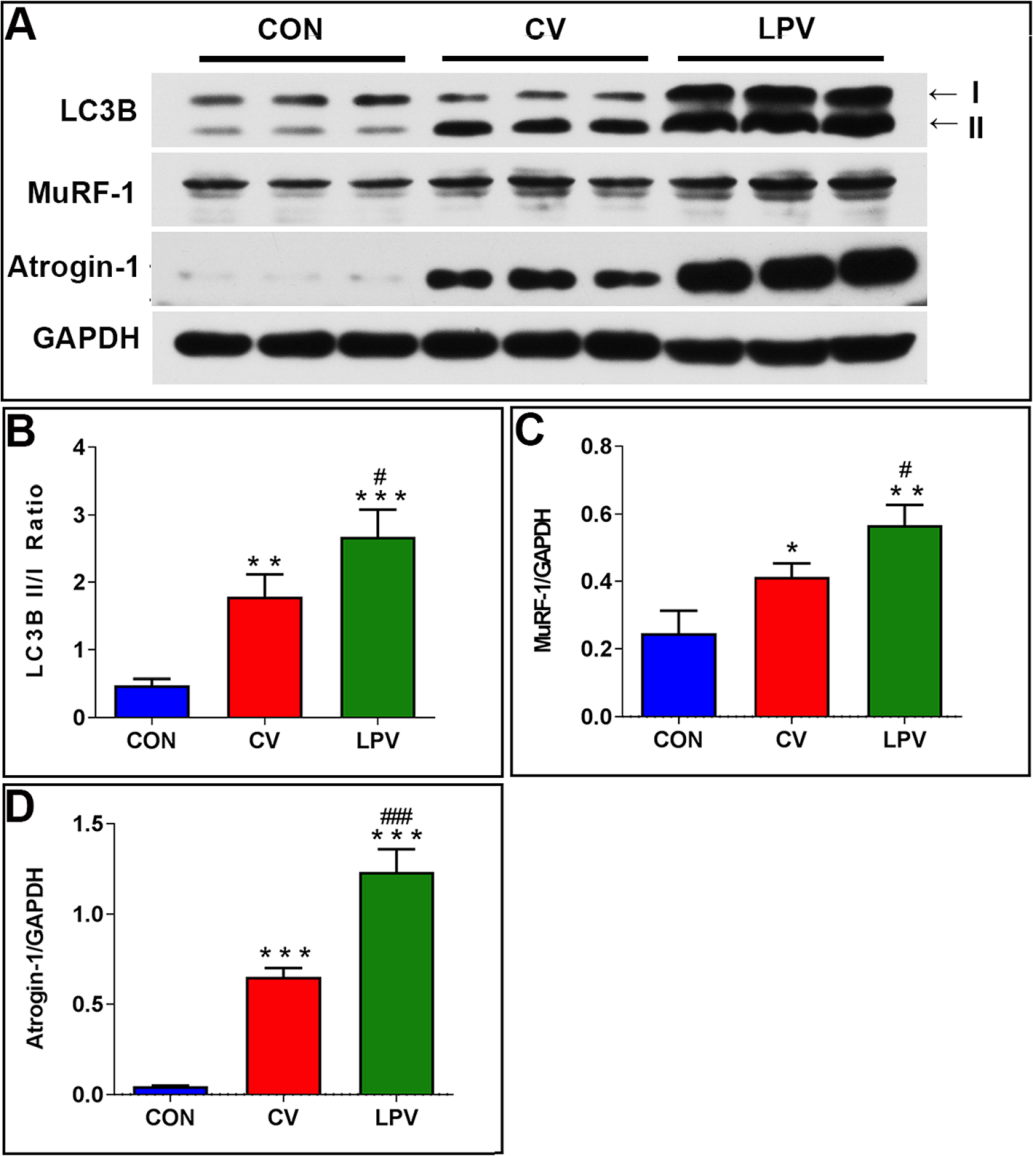
LPV promoted protein degradation in the diaphragm. (**a**), western blots for atrophy markers LC3B–I/II, MuRF–1 and Atrogin–1; The LC3B–II/I ratio (**b**), MuRF–1 (**c**) and Atrogin–1 (**d**) expressions in the LPV and the CV groups were significantly higher than that in the CON group; In addition, LPV increased the protein expressions of those atrophic markers as compared with the CV strategy. *p < 0.05, **p < 0.01, ***p < 0.001 vs. CON group; ^#^*p* < 0.05, ^###^*p* < 0.001 vs. CV group

**Fig. 4:**
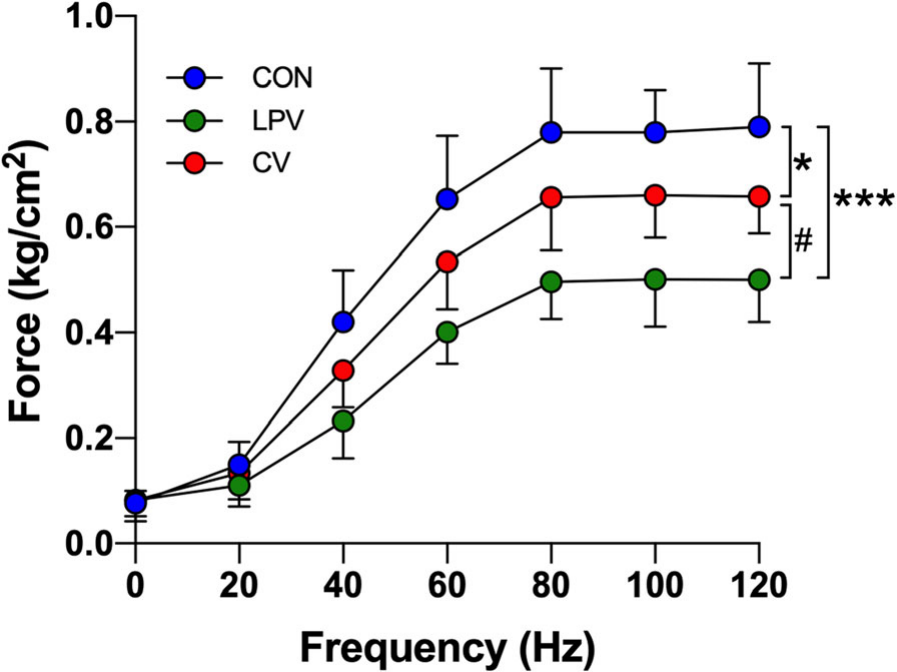
LPV decreased diaphragm forces. The frequency–force curve demonstrates decreased muscle forces in the LPV and the CV group as compared with the CON group. In addition, diaphragm forces in the LPV group were significantly lower than that in the CV group. *p < 0.05, ***p < 0.001 vs. CON group; ^#^*p* < 0.05 vs. CV group

As shown in Fig. 2, the CSA of either slow–twitch (*p* = 0.015) or fast–twitch (*p* = 0.008) fibers in the LPV group were significantly decreased compared with that in the CV group. Western blots showed that atrophic gene expression (LC3B–II/I ratio, Atrogin–1, MuRF–1) was significantly higher in the LPV group than in the CV group (*p* < 0.05) (Fig. 3). In addition, the frequency–force curve demonstrated decreased muscle forces in the LPV group compared with those in the CV group (*p* < 0.05) (Fig. 4). Moreover, the LPV and CV groups showed a higher level of atrophic gene expression and a lower level of fiber CSA and contractile forces than those in the CON group, indicating diaphragm atrophy and weakness in animals receiving LPV or CV.

### Inflammation and oxidative stress in injured lungs and diaphragms

**Fig. 5:**
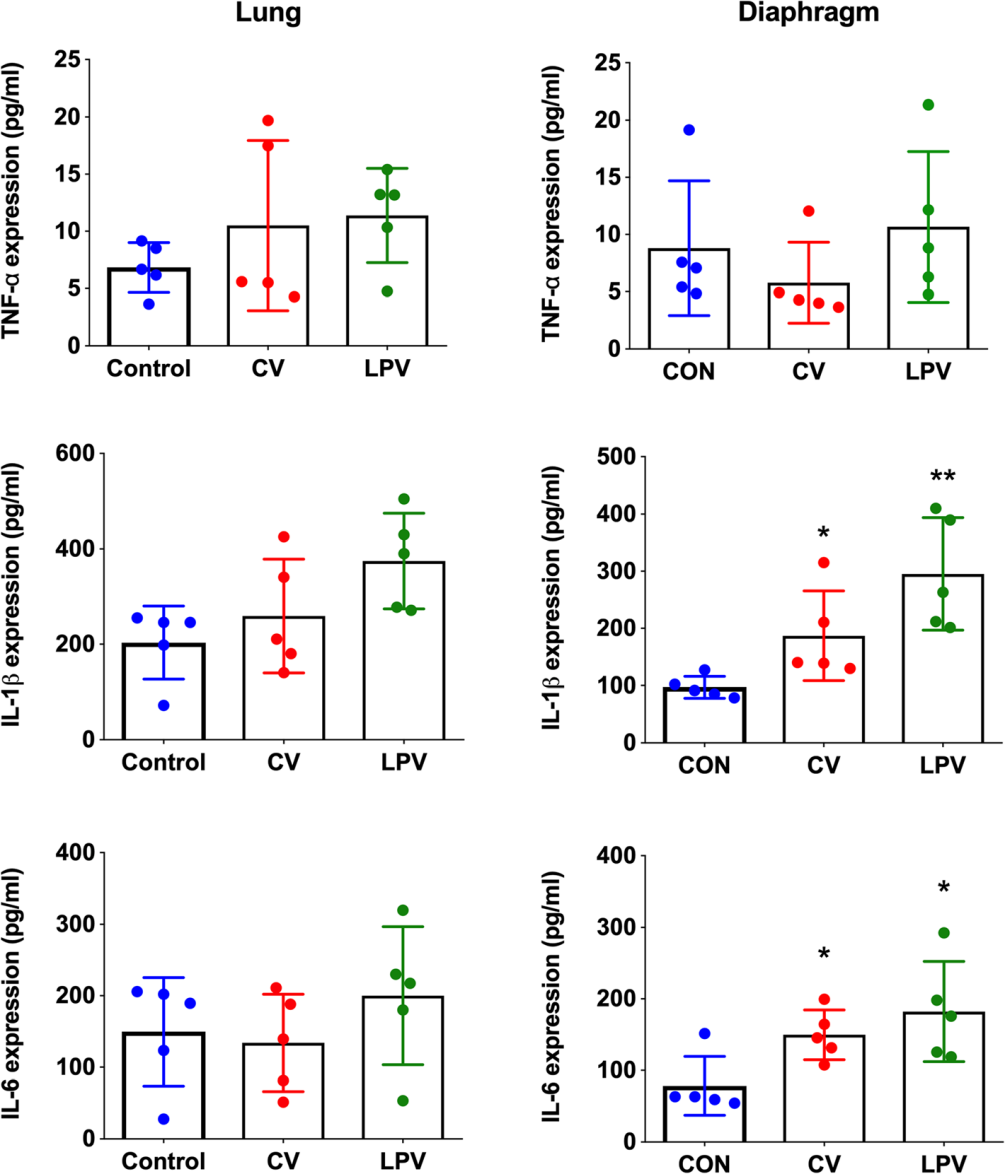
Inflammation in injured lungs and diaphragms. There were no significant differences in pulmonary inflammatory cytokine expressions between the CV and the LPV groups. Diaphragmatic TNF–α expressions were similar between groups, whereas the IL–β and IL–6 expressions were significantly increased in the CV and LPV groups as compared with the CON group. However, no significant differences in the IL–β and IL–6 expressions were observed between the CV and the LPV groups. *p < 0.05, **p < 0.01 vs. CON group

**Fig. 6:**
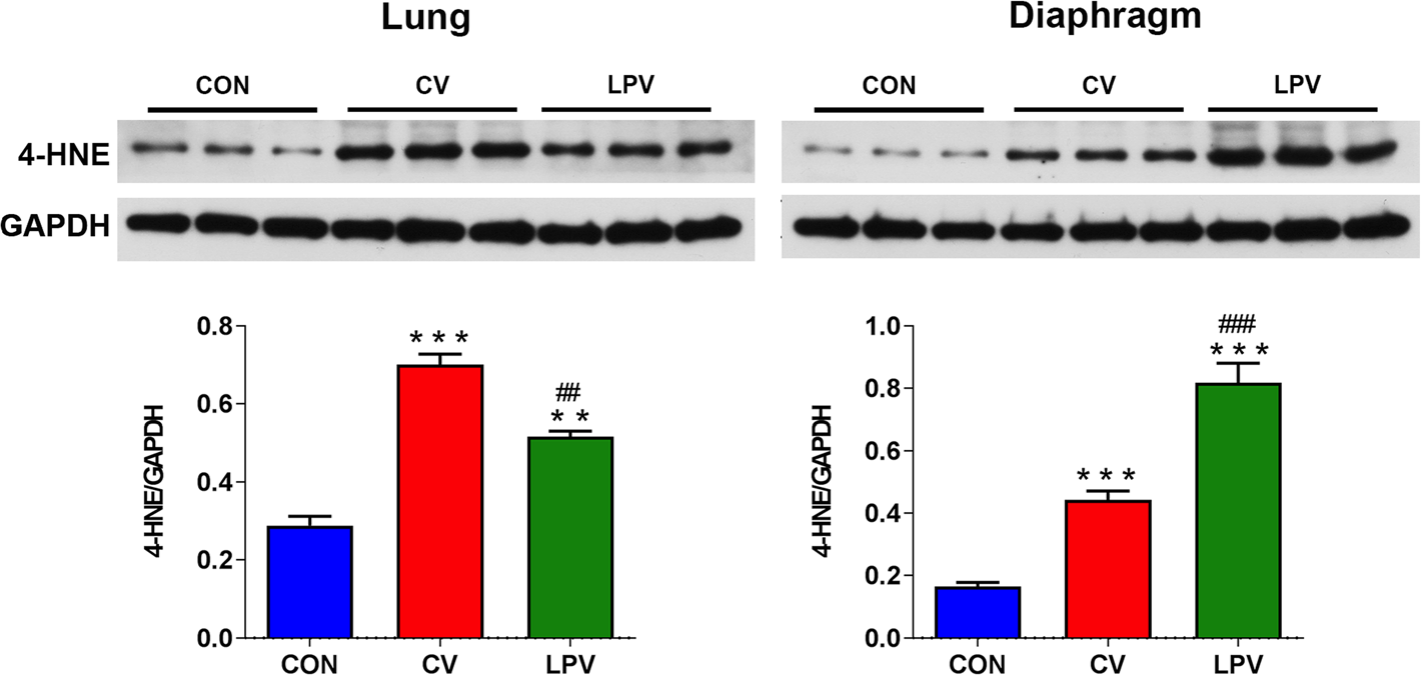
Oxidative stress in injured lungs and diaphragms. Western blots for 4–HNE in injured lungs and diaphragms; our results indicated that pulmonary oxidative stress is downregulated, whereas diaphragmatic oxidative stress is upregulated in the LPV group as compared with the CV group. **p < 0.01, ***p < 0.001 vs. CON group; ^##^*p* < 0.01, ^###^*p* < 0.001 vs. CV group

**Fig. 7:**
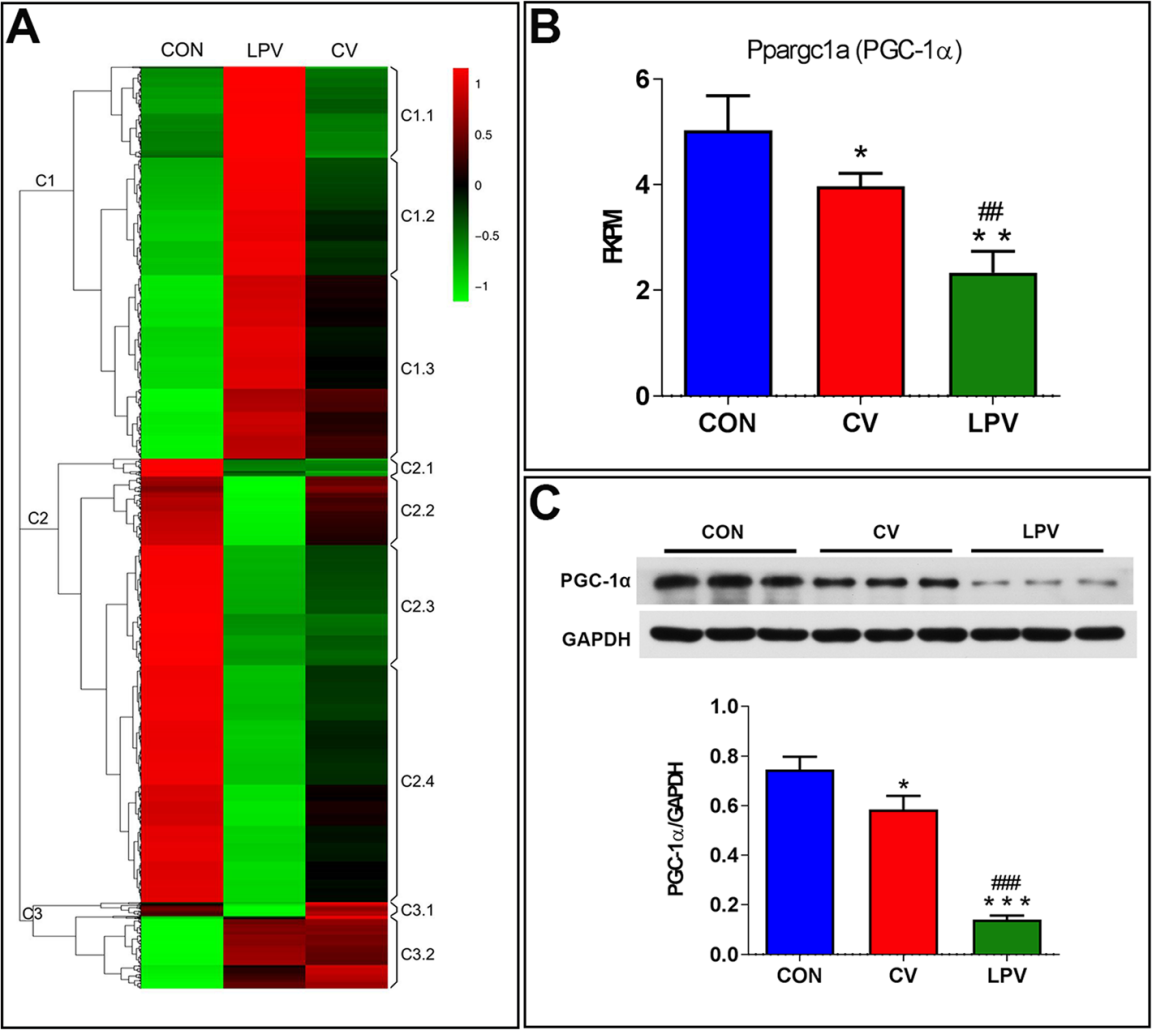
LPV downregulated PGC–1α expressions in the diaphragm. (**a**), the heatmap is generated by selecting differentially expressed genes. These genes could be separated into three major cluster (C1, C2, and C3). The C1 cluster contains up–regulated genes in LPV that are reversed in CV; the C2 cluster contains down–regulated genes in LPV that are mostly reversed in CV except C2.1 which contains down–regulated genes in both LPV and CV; the C3 cluster contains down–regulated genes in LPV that are overly reversed (C3.1, higher than CON) and up–regulated genes in both LPV and CV; (**b**), fold changes in Ppargc1a (PGC–1α) gene expression between the LPV and CV groups; (**c**), western blots for PGC–1α protein expressions in the diaphragm. RNA–seq and western blots suggested that PGC–1α protein expressions were significantly decreased in the LPV group as compared with the CV group. *p < 0.05, **p < 0.01, ***p < 0.001 vs. CON group; ^##^*p* < 0.01, ^###^*p* < 0.001 vs. CV group

As shown in Fig. 5, we found no significant differences in either pulmonary or diaphragmatic cytokine expression between the LPV and CV groups (*p* > 0.05). However, the diaphragmatic expressions of IL–1β and IL–6 were significantly higher in the LPV and CV groups than in the CON group (*p* < 0.05, respectively). Compared to the controls, 4–HNE was elevated in the lungs and the diaphragms of both the CV and LPV groups, but the levels were higher in the lungs and lower in the diaphragm in the CV group than in the LPV group (Fig. 6). As shown in Fig. 7 and Additional file 2: Figure S1, RNA–seq suggested that PGC–1α mRNA expression was significantly decreased in the LPV and CV groups compared with that in the CON group (*p* < 0.05, respectively). In addition, the PGC–1α mRNA expression in the LPV group was significantly lower than that in the CV group (*p* < 0.05). Similar changes in protein expression were detected by western blots.

## Discussion

The major findings of this study can be summarized as follows: (1) the LPV strategy diminished VILI compared with the effect of the CV strategy, (2) LPV increased protein degradation and worsened diaphragm atrophy and weakness in healthy rats with increased oxidative stress compared with the effects of the CV strategy, and (3) the differentially expressed PGC–1α possibly contributed to the different levels of oxidative stress in the diaphragm of animals receiving CV or LPV.

The implementation of the LPV strategy has been shown to diminish VILI in previously non–injured lungs in the perioperative period in the ICU [32]. In the present study, our results showed that the LPV strategy with low V_T_/high PEEP induced less lung injury than the CV strategy, which was in line with previous studies. However, this study showed that in contrast to our hypothesis, LPV worsens VIDD compared with the effect of the CV strategy. It has been reported that the upregulation of inflammatory cytokine expression in the diaphragm is involved in MV–induced diaphragm atrophy [33, 34]. Indeed, inflammation is a potent trigger of skeletal muscle atrophy. TNF–α and other proinflammatory cytokines are able to induce suppression of protein synthesis [35, 36] and upregulation of protein degradation [37] in skeletal muscle. On the other hand, ventilation with low V_T_ was able to attenuate excessive systemic and remote organ inflammation and to preserve nonpulmonary organ (cardiac and kidney) function in animals [38]. Our results showed that 12–h MV with either LPV or CV was able to induce significant increases in diaphragmatic expression of IL–1β and IL–6 but not TNF–α. In addition, no significant differences in cytokine expression were observed between the CV and LPV groups. These data suggested that inflammatory cytokines are not the major contributor to the worsened diaphragm dysfunction in rats.

Oxidative stress serves as a key upstream regulator of proteolysis and the subsequent muscle atrophy that contributes to diaphragm weakness, which then plays a dominant role in the development of VIDD [39, 40]. Surprisingly, we found that the diaphragmatic level of oxidative stress was significantly increased in the LPV group compared with that in the CV group. This can probably explain why LPV worsened VIDD. However, the mechanism by which LPV upregulates oxidative stress in the diaphragm is uncertain. Our results demonstrated that PGC–1α expression in the diaphragm was significantly lower in the LPV group than in the CV and CON groups. PGC–1α is a common and powerful reactive oxygen species (ROS) inhibitor [41], and it has been well demonstrated that PGC–1α plays essential and diverse functions in the control of metabolism and muscle fiber–type switching [42]. Specifically, the expression of PGC–1α is critical for the development of oxidative stress in disused skeletal muscle [43]. In addition, the overexpression of PGC–1α was able to downregulate disuse–induced oxidative stress [44] and attenuate skeletal muscle disuse atrophy [45, 46]. Therefore, it is reasonable to speculate that LPV probably worsens VIDD through the downregulation of diaphragm PGC–1α expression. Previous studies have demonstrated that the inhibition of oxidative stress improves diaphragm function after MV [12, 13]. However, there was no direct evidence indicating that PGC–1α inhibits oxidative stress in the diaphragm during MV. Therefore, further studies should be performed to identify whether enhanced PGC–1α expression is able to attenuate VIDD through the inhibition of oxidative stress in the diaphragm.

According to the current evidence, a high level of V_T_ seems to be beneficial for the diaphragm during ventilation. It has been reported that diaphragm atrophy was associated with profound reductions in V_T_ [47], and extremely large V_T_ ventilation (V_T_: 35 ml/kg; PEEP: 0 cm H_2_O) reduced oxidative stress in the diaphragm compared with the effect of moderate V_T_ ventilation (V_T_: 9 ml/kg; PEEP: 5 cm H_2_O) [48]. However, the effects of PEEP levels on diaphragm functions are uncertain. Some researchers found that high levels of PEEP can preserve diaphragmatic contractility following MV in animals [49]. Very recently, Lindqvist J and colleagues reported that MV with PEEP results in longitudinal atrophy of diaphragm fibers [18], but it is unknown whether this kind of muscle atrophy is also associated with upregulated oxidative stress. However, these findings suggest that high PEEP is a potential risk factor for the development of diaphragm atrophy during MV. In this study, our results showed that the LPV strategy, with low V_T_ and high PEEP, is more harmful to the diaphragm than the CV strategy, with high V_T_ and low PEEP. Collectively, from our observations and those of previous studies, one major problem arises: the LPV strategy that is commonly used in clinical practice probably worsens VIDD compared with the CV strategy.

There are four major limitations in the present study. First, to avoid systemic hypoxia, we adjusted the ventilation rate and oxygen supply according to arterial blood gas analysis. However, whether hypoxia can be avoided in the CV group when MV exceeds 12 h is questionable. Once the adjustment of the ventilation rate and oxygen supply is insufficient to maintain appropriate pulmonary gas exchange due to massive damage to the lungs, systemic hypoxia can induce excessive generation of ROS and affect nonpulmonary organs, including the diaphragm. In addition, the LPV strategy is preferable to induce hypercapnia compared with high V_T_/low PEEP ventilation [17], and hypercapnia has been shown to protect the diaphragm against ventilator–induced atrophy and weakness [16]. Therefore, whether the LPV strategy can protect the diaphragm against VIDD when MV exceeds 12 h or in the absence of ventilator adjustment needs to be explored. Second, we used a CV strategy with high V_T_ and low PEEP as a positive control in this study. However, this kind of ventilation strategy is rarely used in current clinical practice. In addition, we compared these two different strategies (LPV vs. CV) instead of separately evaluating the effects of different levels of V_T_ or PEEP on diaphragm function during MV. Therefore, it is uncertain whether the low V_T_ or the high PEEP worsens diaphragm muscle atrophy and weakness compared with the effect of the CV strategy. However, our data revealed that the LPV strategy diminished VILI but worsened VIDD. Third, our data suggested that the downregulation of PGC–1α could be a possible contributor to the LPV–induced oxidative stress in the diaphragm, but our data are limited to support its roles in the development of VIDD. Further studies focusing on VIDD in PGC–1α-overexpressing and knockout animals should be performed. Finally, a human blood gas analyzer was used to determine rat blood gases in the present study. However, the reliability of this instrument has not yet been well determined in animals.

## Conclusions

In clinical practice, LPV with low V_T_ and high PEEP is the most important ventilation strategy because its lung–protective effects markedly improve respiratory mechanics and patient outcomes. We first report that the LPV strategy with low V_T_ and high PEEP worsens diaphragm dysfunction compared with the effects of the CV strategy with high V_T_ and low PEEP. Since diaphragm dysfunction is tightly associated with weaning failure and the poor outcomes of ventilated patients, diaphragm-protecting ventilation should also be considered. Together, our results suggest that the LPV strategy worsens ventilator–induced diaphragm atrophy and weakness by inducing oxidative stress, probably through the downregulation of PGC–1α in the diaphragm. Therefore, finding a ventilation strategy with appropriate tidal volume and PEEP for the simultaneous protection of the lungs and diaphragm is challenging and urgent.

## Supplementary information

**Additional file 1: Table S1.** Damage severity distribution in all groups

**Additional file 2: Figure S1.** RNA-seq results.

## Data Availability

The data are available from the corresponding author on reasonable request.

## Abbreviations

CSA: Cross–sectional area
CV: Conventional ventilation
LPV: Lung–protective ventilation
PEEP: Positive end–expiratory pressure
PGC–1α: Peroxisome proliferator–activated receptor γ coactivator–1alpha
ROS: Reactive oxygen species
VIDD: Ventilator–induced diaphragm dysfunction
VILI: Ventilator–induced lung injury
V_T_: Tidal volume
