# Supplementary material for: Lung-protective ventilation worsens ventilator-induced diaphragm atrophy and weakness
Source: Respir Res. 2020 Jan 10;21:16. doi: 10.1186/s12931-020-1276-7 (PMC6954632; doi:10.1186/s12931-020-1276-7)
Supplement: Supplementary file 1 — Additional file 1: Table S1. Damage severity distribution in all groups [file 12931_2020_1276_MOESM1_ESM.docx]

**Table S1. Damage severity distribution in all groups**

|  | **CON (n = 5)** | **CV (n = 5)** | **LPV (n = 5)** |
| --- | --- | --- | --- |
| **Alveolar wall injury** |  |  |  |
| Mild/no (≤1) | 0 (0) | 0 (0) | 2 (40%) |
| Moderate (2) | 0 (0) | 2 (40%) | 3 (60%) |
| Severe/maximal (≥3) | 0 (0) | 3 (60%) | 0 (0) |
| **Inflammatory cell infiltration** |  |  |  |
| Mild/no (≤1) | 1(100%) | 0 (0) | 4 (80%) |
| Moderate (2) | 0 (0) | 1 (20%) | 1 (20%) |
| Severe/maximal (≥3) | 0 (0) | 4 (80%) | 0 (0) |
| **Hemorrhage** |  |  |  |
| Mild/no (≤1) | 0 (0) | 1 (20%) | 3 (60%) |
| Moderate (2) | 0 (0) | 1(20%) | 1 (20%) |
| Severe/maximal (≥3) | 0 (0) | 3 (60%) | 1 (20%) |

Data are expressed as number and percentages, and the comparison of percentages were performed using Fisher’s exact test. The percentage of severe/maximal damages in alveolar wall thickness, inflammatory cell infiltration and hemorrhage in the CV group were significantly higher than that in the LPV group (*p* < 0.05, respectively). In addition, the percentage of severe/maximal damage for hemorrhage in both the CV and LPV groups were higher than that in the CON group (*p* < 0.05, respectively).
