# Supplementary figures and images for: Lung-protective ventilation worsens ventilator-induced diaphragm atrophy and weakness
Source: Respir Res. 2020 Jan 10;21:16. doi: 10.1186/s12931-020-1276-7 (PMC6954632; doi:10.1186/s12931-020-1276-7)

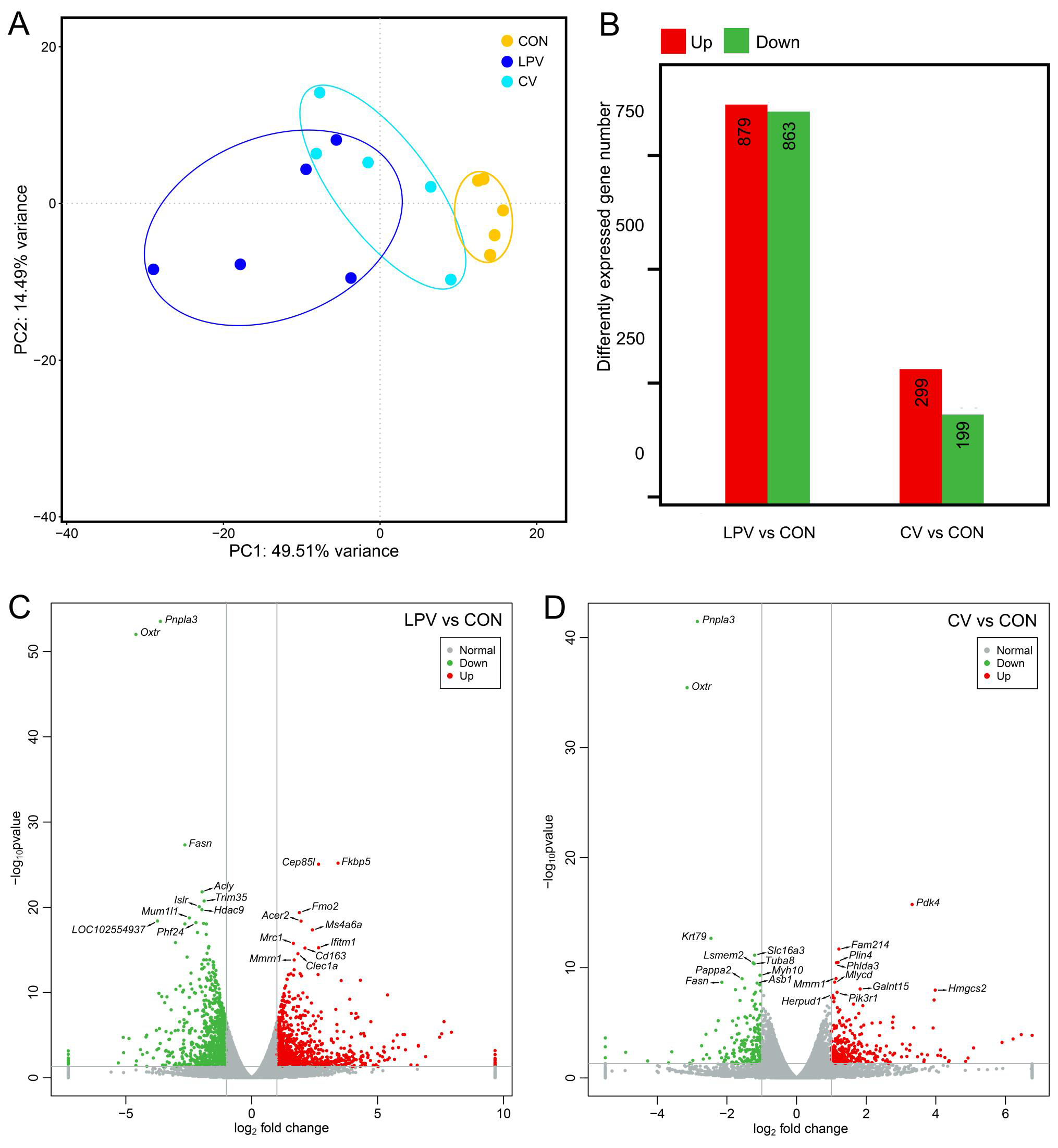

Supplement: Supplementary file 2 — Additional file 2: Figure S1. RNA-seq results. [file 12931_2020_1276_MOESM2_ESM.tif]
